# Hemotrophic mycoplasma in Simmental cattle in Bavaria: prevalence, blood parameters, and transplacental transmission of ‘*Candidatus* Mycoplasma haemobos’ and *Mycoplasma wenyonii*

**DOI:** 10.1186/s13028-018-0428-y

**Published:** 2018-11-16

**Authors:** Florian Martin Niethammer, Julia Ade, Ludwig Eduard Hoelzle, Benjamin Schade

**Affiliations:** 1 Tiergesundheitsdienst Bayern e.V., Senator-Gerauer-Str. 23, 85586 Poing, Germany; 2 Institut für Nutztierwissenschaften, Universität Hohenheim, Garbenstr. 30, 70593 Stuttgart, Germany

**Keywords:** Acridine-orange-stained blood smears, Anemia, Blood parameters, ‘*C.* M. haemobos’, Cattle, Hemotrophic mycoplasma, *M. wenyonii*, Prevalence, Real-time PCR, Vertical transmission

## Abstract

**Background:**

The significance of hemotrophic mycoplasma in cattle remains unclear. Especially in Europe, their epidemiological parameters as well as pathophysiological influence on cows are lacking. The objectives of this study were: (1) to describe the prevalence of ‘*Candidatus* Mycoplasma haemobos’ (‘*C.* M. haemobos’) and *Mycoplasma wenyonii* (*M. wenyonii*) in Bavaria, Germany; (2) to evaluate their association with several blood parameters; (3) to explore the potential of vertical transmission in Simmental cattle; and (4) to evaluate the accuracy of acridine-orange-stained blood smears compared to real-time polymerase chain reaction (PCR) results to detect hemotrophic mycoplasma. A total of 410 ethylenediaminetetraacetic acid-blood samples from cows from 41 herds were evaluated by hematology, acridine-orange-stained blood smears, and real-time PCR. Additionally, blood samples were taken from dry cows of six dairy farms with positive test results for hemotrophic mycoplasma to investigate vertical transmission of infection.

**Results:**

The period prevalence of both species was 60.24% (247/410), *C.* M. haemobos 56.59% (232/410), *M. wenyonii* 8.54% (35/410) and for coinfection 4.88% (20/410). Of the relevant blood parameters, only mean cell volume (MCV), mean cell hemoglobin (MCH), and white blood cell count (WBC) showed differences between the groups of infected and non-infected individuals. There were lower values of MCV (P < 0.01) and MCH (P < 0.01) and higher values of WBC (P < 0.05) in ‘*C.* M. haemobos’-infected cows. In contrast, co-infected individuals had only higher WBC (P < 0.05). In *M. wenyonii*-positive blood samples, MCH was significantly lower (P < 0.05). Vertical transmission of ‘*C.* M. haemobos’ was confirmed in two calves. The acridine-orange-method had a low sensitivity (37.39%), specificity (65.97%), positive predictive value (63.70%) and negative predictive value (39.75%) compared to PCR.

**Conclusions:**

‘*Candidatus* Mycoplasma haemobos’ was more prevalent than *M. wenyonii* in Bavarian Simmental cattle, but infection had little impact on evaluated blood parameters. Vertical transmission of the infection was rare. Real-time PCR is the preferred diagnostic method compared to the acridine-orange-method.

## Background

*Mycoplasma wenyonii* (*M. wenyonii*) [1–3] and the recently discovered, not yet fully classified, species ‘*Candidatus* Mycoplasma haemobos’ (‘*C.* M. haemobos’) [4, 5] have been described as major hemotrophic mycoplasma in cattle. Hemotrophic mycoplasma infections have been associated with various unspecific clinical signs and resulting clinical relevance. Decline in milk yield, reproductive inefficiency, weight loss, transient fever, rough hair coat, vaginal bleeding, regenerative anemia, icterus, and reduced body weight at birth of infected calves were associated with a single infection or coinfection of *M. wenyonii* and ‘*C.* M. haemobos’ [6–12]. Some studies did not find any correlation between an infection with either ‘*C.* M. haemobos’ or *M. wenyonii* and the occurrence of regenerative anemia or changes of various blood parameters in cattle [6, 10, 11, 13, 14]. In contrast, coinfection with *Anaplasma (A.) marginale* was shown to enhance anemia and to be potentially fatal [15, 16]. However, infection with bovine leukemia virus was unlikely to be a risk factor. Both hemotrophic mycoplasmas have been described to be transmitted either horizontally by insects or vertically/transplacentally [13, 17, 18]. Among described risk factors, cattle in indoor housing and cattle aged 1–3 years seem to be more likely to get infected than pasture-based or older cattle, respectively [6].

The prevalence of infection varies by study and country. A recent Japanese study found a prevalence of 69.4% in 36 cows in Hiroshima and 93.8% in 32 cows in Miyazaki [19]. Another larger Japanese study with 343 cows also found a 64.7% prevalence with hemotrophic mycoplasmas—almost evenly attributed to *M. wenyonii* (38.5%) and ‘*C.* M. haemobos’ (39.1%) [20]. Similarly, a large Brazilian study with 433 cows described a prevalence of ‘*C.* M. haemobos’ of 60.97% [21].

The diagnosis of hemotrophic mycoplasma infection has traditionally been made by the visual assessment of acridine-orange-stained blood smears with fluorescence microscopy [22]. Here, contrast-stained, dot-like deposits on erythrocytes are visible. However, the risk of misinterpretation of artifacts or other infections is high [23, 24]. Therefore, hemotrophic mycoplasmas are currently identified with either 16S rDNA polymerase chain reaction (PCR) [25] or real-time PCR [26]. The latter seems to be superior to the former in cats [27] and cattle [28], but confirmative data are lacking.

Due to the limited information of the infection in Bavarian cattle, the objectives of this study were (1) determine the prevalence of ‘*C.* M. haemobos’ and *M. wenyonii* in Bavarian Simmental cattle, (2) evaluate the association of infection with hematological parameters, (3) evaluate the likelihood of transplacental transmission, and (4) evaluate the accuracy of detecting hemotrophic mycoplasma by acridine-orange-stained blood smears compared to real-time PCR results.

## Methods

**Fig. 1:**
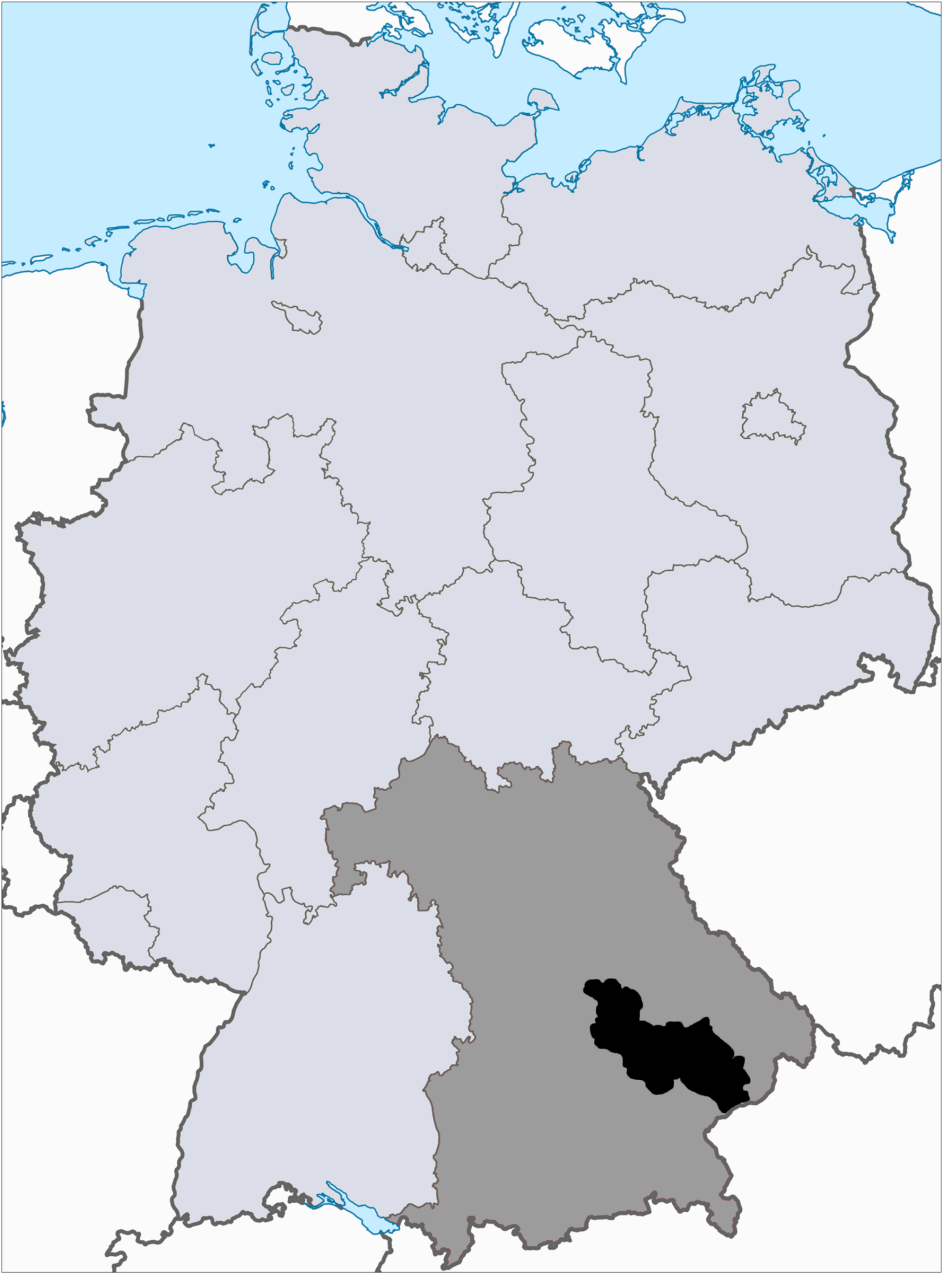
Geographical location of sample origin in Germany. Grey area: Bavaria; Black area: origin of blood samples (administrative districts Kehlheim, Landshut, Dingolfing-Landau and Rottal-Inn); map based on: Wikimedia Commons, Germany location map.svg

A convenience sample of 41 dairy farms from the four South-Eastern-Bavarian administrative districts Kehlheim, Landshut, Dingolfing-Landau, and Rottal-Inn were enrolled (Fig. 1). Farms were clients of the Bavarian Animal Health Services that were willing to participate. At each farm 9 mL ethylenediaminetetraacetic acid (EDTA) blood samples were taken from 10 adult Simmental dairy cows during routine herd health visits from September 15, 2015 until July 11, 2016. Furthermore, on six farms with a history of hemotrophic mycoplasma infection, EDTA blood samples were collected from dry cows and their calves immediately post natum, prior to the first feeding of colostrum. All samples were delivered immediately to the laboratory by our courier service and were processed within 24 h after drawing of blood, including blood smears.

Each blood sample was analyzed for erythrocyte count (RBC), hemoglobin (Hb), hematocrit (Hct), mean cell volume (MCV), red blood cell distribution width (RDW), mean cell hemoglobin concentration (MCHC), mean cell hemoglobin (MCH), reticulocyte (Retic), platelet as well as white blood cell count (WBC) by automatic blood cell counter (IDEXX ProCyte Dx; IDEXX GmbH, Ludwigsburg, Germany).

Additionally, blood smears were stained with acridine orange (Carl Roth GmbH & Co. KG, Karlsruhe, Germany). Briefly, blood smears were fixed with ethanol for 1 min and then air dried. Acridine orange solution (0.25 mg/mL) was applied for 30 min in the dark. Subsequently, the slides were rinsed with water, air dried, and evaluated under ultraviolet (UV) light excitation with a fluorescence microscope for erythrocytes infected with hemotrophic mycoplasma. Furthermore, aliquots of the blood samples were tested by quantitative real-time PCR as described previously [28]. Briefly, for DNA extraction, 200 µL of EDTA-anticoagulated blood was mixed with an equal volume of lysis buffer (10 mM Tris pH 7.5, 5 mM MgCl_2_, 330 mM sucrose, 1% (v/v) Triton X-100) and centrifuged (8000×*g*, 21 °C, 1 min). DNA was purified from the pellet using the GenElute™ Bacterial Genomic DNA Kit (Sigma-Aldrich, Steinheim, Germany). To determine cross reaction, one PBS control was included in each extraction run (10 samples). *‘C*. M. haemobos’ and *M. wenyonii* were quantitatively detected with the StepOne™ System (Applied Biosystems, Foster City, USA). The reaction mixture (20 µL) contained 10 µL of the 2× SYBR Green PCR Master Mix (Thermo Fisher, Munich, Germany), 0.5 µM of primer each, and 2 µL of template DNA. Dilutions of plasmid DNA defining 10^5^, 10^4^, 10^3^ genome equivalents (GE) per reaction were included as standards into the runs.

Based on the results of the real-time PCR, cattle were categorized into ‘infection negative’ (no detection of ‘*C.* M. haemobos’ or *M. wenyonii*), ‘infection total’ (detection of either ‘*C.* M. haemobos’ or *M. wenyonii*), ‘*C.* M. haemobos’ (only detection of ‘*C.* M. haemobos’), *M. wenyonii* (only detection of *M. wenyonii*) and ‘coinfection’ (detection of both ‘*C.* M. haemobos’ and *M. wenyonii*).

All data were recorded in Microsoft EXCEL Microsoft Home and Student 2010, Version 14.0.7208.5000 (32 Bit), Microsoft Corporation 2010 and statistically analyzed in R× 64 Version 3.3.1 and RStudio Version 1.0.136 enhanced by packages “caret” and “e1071”. Level of significance was defined as α = 0.05.

Prevalence was calculated from the whole dataset of 410 samples. Statistical analysis of every other parameter but blood stains were calculated from a dataset of 409 samples. No blood cell count results were available in one sample due to technical problems.

The Shapiro–Wilks-test was performed to test for normal distribution of subgroups ‘infected’ or ‘non-infected’ for hematological parameters and indices. If normal distribution in both subgroups was confirmed, homoscedasticity was examined by Bartlett’s-test. If normal distribution was rejected for one or both subgroups, Fligner-Killeen’s-test was calculated. If there was no homogeneity of variances in normally distributed data, Welch’s *t* test was calculated. Whether or not infection with hemotrophic mycoplasmas was associated with hematological parameters was determined by computing Student’s or Welch’s independent two-sample-t-test if both subgroups were normally distributed. If one or both subgroups were not normally distributed, Wilcoxon’s rank-sum-test for independent samples was performed. In order to visualize relevant results, boxplots were generated, and its quantiles and medians were shown in numbers.

To assess intrauterine transmission, data was analyzed by Fisher’s-exact-test. The test was computed for blood samples positive for ‘*C.* M. haemobos’ or *M. wenyonii* separately. If identical species were demonstrated in corresponding blood-samples of cows and calves, transplacental transmission of hemotrophic mycoplasma in cattle was assumed.

The accuracy of detecting hemotrophic mycoplasmas via acridine-orange-stained blood smears was evaluated with a reduced dataset containing only 374 blood samples from 38 dairy farms, since no acridine-orange-stained blood smear evaluation was performed in the missing 36 blood samples due to laboratory problems. Reference values for this were generated by real-time PCR-testing. To specify the validation of the acridine-orange-method by the real-time PCR test-results, sensitivity, specificity, positive predictive value, and negative predictive value were determined compared to the real-time PCR as a gold standard by McNemar’s test and Cohen’s Kappa.

## Results

Without accounting for herd, 60.24% (247/410) of cows tested positive by real-time PCR at least once for hemotrophic mycoplasma. More specifically, 56.59% (232/410) of cows were positive for ‘*C.* M. haemobos’, while 8.54% (35/410) and 4.88% (20/410) were positive for *M. wenyonii* or mixed infections, respectively. ‘*C.* M. haemobos’ was found in all tested herds (n = 41), while *M. wenyonii* was only detected in 16 of 41 farms. In comparison with the PCR detection, acridine-orange-stained blood smears showed a sensitivity of 37.39%, a specificity of 65.97%, a positive predictive value of 63.70%, and a negative predictive value of 39.75%. One hundred thirty-five blood smears were positive and 239 were negative for hemotrophic mycoplasma in contrast to 144 and 230 by real-time PCR, respectively. McNemar’s test P-value was  <  0.0001 and Cohen’s Kappa was 0.0299.

**Fig. 2:**
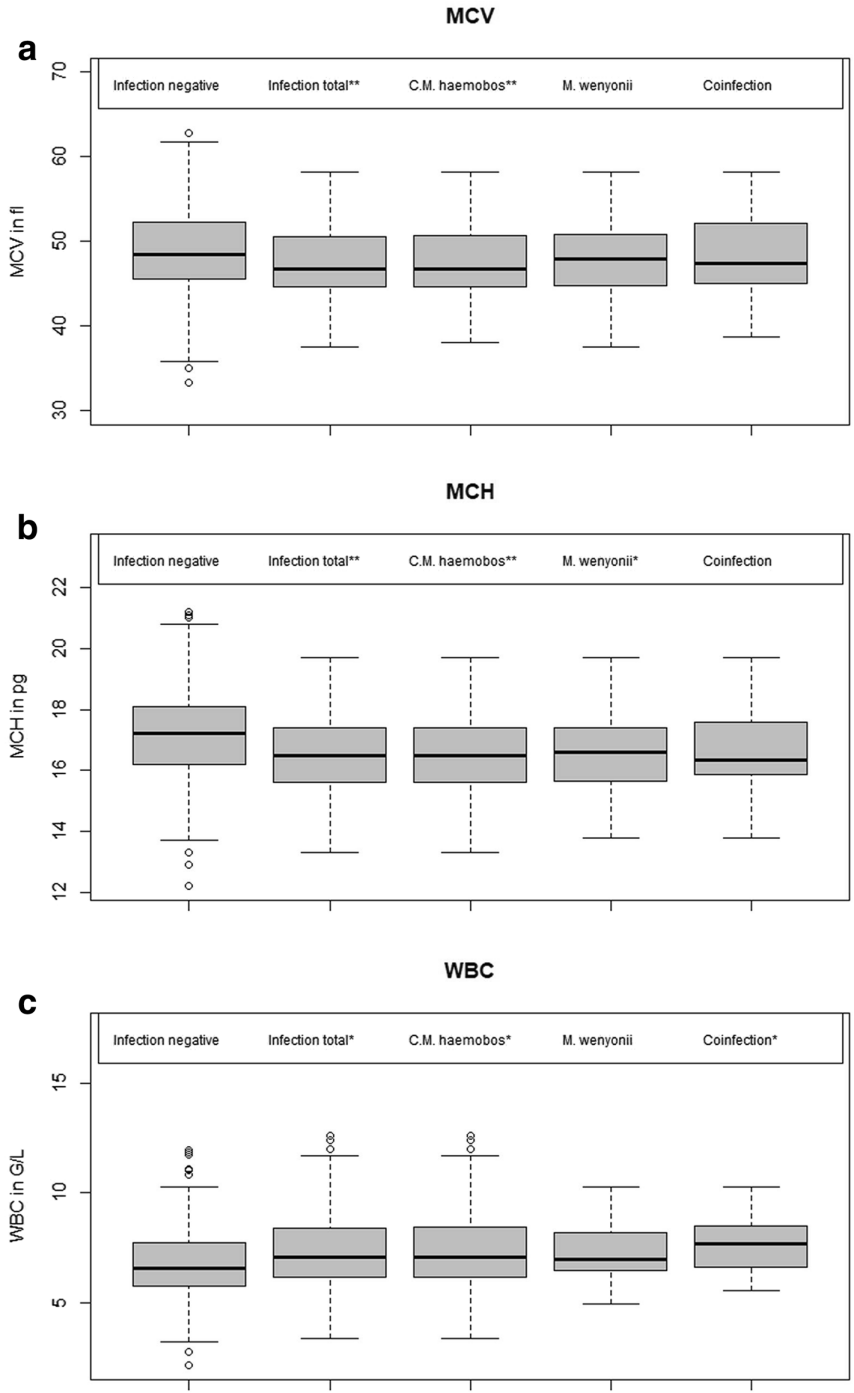
Statistical analysis and comparison of selected blood parameters. Hematological results of 410 EDTA blood samples of Simmental cows and their distribution are shown using boxplot analysis. Groups were classified by real-time PCR results. **a** Mean cell volume (MCV) values, **b** mean cell hemoglobin (MCH) values, **c** white blood cell count (WBC) values. **Group differs from group “Infection negative” with P < 0.01. *Group differs from group “Infection negative” with P < 0.05

No differences were found for Hct, Hb, Retic, RBCs, and MCHC between infected and non-infected cows. ‘*C.* M. haemobos’ real-time-PCR positive cows showed lower values for MCV (P < 0.01), MCH (P < 0.01) and higher values for WBC (P < 0.05) (Fig. 2a–c, Table 1) while cows positive for *M. wenyonii* had lower MCH than ‘infection negative’ individuals (P < 0.05) (Fig. 2a, Table 1). In co-infected animals, only WBC was higher than in ‘infection negative’ individuals (P < 0.05) (Fig. 2c, Table 1).

**Table 1:** Hematological parameters of infected and non-infected cows

Parameter	Infection negative (n = 163)	Infection total (n = 246)	*‘Candidatus* Mycoplasma haemobos’ (n = 231)	*Mycoplasma wenyonii* (n = 35)	Co-infection (n = 20)
Hct (%)					
Median	28.20	27.50	27.60	27.10	28.85
2. Quartile	25.95	25.50	25.50	26.10	26.90
4. Quartile	30.25	29.70	29.80	29.65	30.30
Hb (g/dL)					
Median	9.9	9.65	9.7	9.60	9.95
2. Quartile	9.1	9.00	9.0	9.15	9.40
4. Quartile	10.5	10.40	10.4	10.30	10.40
Retic (× 10^3^/µL)					
Median	3.50	3.5	3.5	3.60	4.1
2. Quartile	2.70	2.6	2.6	2.60	3.2
4. Quartile	4.65	4.7	4.8	4.35	5.4
RBC (× 10^6^/µL)					
Median	5.70	5.84	5.84	5.81	5.86
2. Quartile	5.35	5.43	5.44	5.38	5.41
4. Quartile	6.25	6.32	6.33	6.19	6.33
MCV (fL)					
Median	48.50	46.8**	46.70**	48.00	47.45
2. Quartile	45.60	44.6	44.60	44.85	45.00
4. Quartile	52.35	50.6	50.65	50.80	52.20
MCH (pg)					
Median	17.2	16.5**	16.5**	16.60*	16.35
2. Quartile	16.2	15.6	15.6	15.65	15.85
4. Quartile	18.1	17.4	17.4	17.40	17.60
MCHC (g/dL)					
Median	34.9	35.1	35.10	34.80	34.60
2. quartile	34.1	34.2	34.20	34.00	33.65
4. quartile	35.8	36.0	35.95	35.65	35.40
WBC (G/L)					
Median	6.56	7.06*	7.09*	6.96	7.69*
2. Quartile	5.77	6.18	6.18	6.48	6.60
4. Quartile	7.74	8.40	8.46	8.21	8.47

Difference from “infection negative”: * P < 0.05; ** P < 0.01

Finally, 50 dry cows were tested for a potential transplacental transmission. Of these, 18 cows (36.0%) were positive for ‘*C.* M. haemobos’, two cows were positive for *M. wenyonii* (4.0%), and seven cows were positive for both ‘*C.* M. haemobos’ and *M. wenyonii* (14.0%). It was possible to take 25 blood samples of corresponding newborn calves. Two of the tested calves were positive for ‘*C.* M. haemobos’ (Table 2)-one was born to a co-infected mother while the other calf came from a PCR-negative cow. All 25 calves were negative for *M. wenyonii*.

**Table 2:** Correlation of infection with *‘Candidatus* Mycoplasma haemobos’ (*‘C.* M. haemobos’) in cows and their calves

Variable	Calves real-time PCR		Total (%)	P-value*	OR*	95% CI*
	Positive (%)	Negative (%)				
Cows real-time PCR						
Positive (%)	1 (4.0)	11 (44.0)	12 (48.0)	1	1.09	0.01 < OR < 92.61
Negative (%)	1 (4.0)	12 (48.0)	13 (52.0)			
Total (%)	2 (8.0)	23 (92.0)	25 (100.0)			

*CI* confidence interval, *OR* odds ratio, *PCR* polymerase chain reaction

* Fisher’s exact test

## Discussion

The study provided data on hemotrophic mycoplasma in Bavarian Simmental cattle for the first time. The total prevalence of 60.24% for hemotrophic mycoplasma in Bavarian Simmentals was similar to that in Brazilian cattle [21] but slightly lower than that reported for Japan (69.4%, and 93.8% [19] and 64.7% [20]). Interestingly, the prevalence of the single species differed from those previously published in Japan, where ‘*C.* M. haemobos’ and *M. wenyonii* were evenly distributed. In contrast, in the current study ‘*C.* M. haemobos’ was much more prevalent than *M. wenyonii* or co-infections. These observed differences could be due to differences in climate, which could favor vector borne transmission, breed susceptibility, different housing systems (barn vs. pasture), or a higher proportion of younger animals in Japan than in Bavaria, but we did not collect specific data to support this hypothesis in this study [17, 20]. Because every sampled herd except one was only barn-housed, tick borne transmission can be neglected as a route of infection, whereas fly bites cannot be excluded.

Furthermore, unlike other studies [6–9, 11, 15, 16, 20], no association between infection status and anemia was observed. Instead, the observations of this study supported the results of other authors, especially for *M. wenyonii*, that also did not find an association [11, 12, 14]. However, there seems to be interaction between hemotrophic mycoplasma and erythrocytes, as both MCV and MCH decreased in the groups ‘infection total’ and ‘*C.* M. haemobos’. Similarly, MCH decreased in the *M. wenyonii* group. The lack of statistical difference in MCV or WBC between *M. wenyonii*’s and co-infected or ‘non-infected’ cows might be due to the small sample size of *M. wenyonii*-infected animals. The WBC increased, at least numerically, showing the highest levels in the group of co-infected cows. This may be a consequence of a presumably stronger stimulation of immunity by coinfection and consequently higher numbers of leukocytes. Future studies need to investigate whether anemia is dependent on co-infection of hemotrophic mycoplasma with other bacteria or on additional external factors, such as breed susceptibility. The observations of this study underlined again the limitations of acridine-orange stained blood smears for the detection of hemotrophic mycoplasma [23, 24, 29]. Since blood smears were made just after arrival of the blood samples at the laboratory, EDTA-caused detachment of hemotrophic mycoplasma from parts of the erythrocyte membranes could have contributed to the above-mentioned limitations in comparison to real-time PCR, by producing false negative results. The results of McNemar’s test and Cohen’s Kappa indicate significant difference between the two methods (acridin-orange-stained blood smears vs. real-time PCR) and poor inter-rater agreement, respectively.

The results also support previously reported vertical transmission of ‘*C.* M. haemobos’ in Simmental cattle. Similarly to another study, one calf that tested positive by real-time PCR for ‘*C.* M. haemobos’ was born from a negatively tested mother in this study [17]. One might speculate that the bacterial load was below the detection threshold of real-time PCR or indeed not present, when the blood sample was collected from the PCR negative dam. The obvious low frequency of transplacental transmission of ‘*C.* M. haemobos’ in cattle suggests that vertical infection is a minor pathway of infection. The low prevalence of *M. wenyonii* did not allow us to make inferences about transplacental transmission of *M. wenyonii* in cattle. Further investigations are needed to clarify this pathway of infection.

## Conclusions

‘*Candidatus* Mycoplasma haemobos’ was more prevalent than *M. wenyonii* in Bavarian Simmental cattle, but an infection had little impact on evaluated blood parameters. Vertical transmission of the infection was observed for ‘*C.* M. haemobos’. Real-time PCR was found to be the preferred diagnostic method over the acridine-orange-detection method.
